# Karyotype relationships among selected deer species and cattle revealed by bovine FISH probes

**DOI:** 10.1371/journal.pone.0187559

**Published:** 2017-11-07

**Authors:** Jan Frohlich, Svatava Kubickova, Petra Musilova, Halina Cernohorska, Helena Muskova, Roman Vodicka, Jiri Rubes

**Affiliations:** 1 Central European Institute of Technology - Veterinary Research Institute, Brno, Czech Republic; 2 Zoo Prague, Prague, Czech Republic; University of Florence, ITALY

## Abstract

The Cervidae family comprises more than fifty species divided into three subfamilies: Capreolinae, Cervinae and Hydropotinae. A characteristic attribute for the species included in this family is the great karyotype diversity, with the chromosomal numbers ranging from 2n = 6 observed in female *Muntiacus muntjak vaginalis* to 2n = 70 found in *Mazama gouazoubira* as a result of numerous Robertsonian and tandem fusions. This work reports chromosomal homologies between cattle (*Bos taurus*, 2n = 60) and nine cervid species using a combination of whole chromosome and region-specific paints and BAC clones derived from cattle. We show that despite the great diversity of karyotypes in the studied species, the number of conserved chromosomal segments detected by 29 cattle whole chromosome painting probes was 35 for all Cervidae samples. The detailed analysis of the X chromosomes revealed two different morphological types within Cervidae. The first one, present in the Capreolinae is a sub/metacentric X with the structure more similar to the bovine X. The second type found in Cervini and Muntiacini is an acrocentric X which shows rearrangements in the proximal part that have not yet been identified within Ruminantia. Moreover, we characterised four repetitive sequences organized in heterochromatic blocks on sex chromosomes of the reindeer (*Rangifer tarandus*). We show that these repeats gave no hybridization signals to the chromosomes of the closely related moose (*Alces alces*) and are therefore specific to the reindeer.

## Introduction

The Cervidae family includes more than fifty different species which are divided into three subfamilies: Capreolinae, Cervinae and Hydropotinae [1]. Several of the Cervidae species have a growing economic potential as farm animals, but they are also hunted for trophies. However, most species are threatened and need protection. A distinctive trait of this family is the great diversity of karyotypes amongst respective species, with the chromosomal numbers (2n) ranging from 6 in female *Muntiacus muntjak vaginalis* to 70 in *Mazama gouazoubira* [2]. The chromosomal evolution in particular cervid subfamilies occurred by different types of rearrangements. For example, Robertsonian fusions are the most common type of chromosomal rearrangements in Cervini, whereas tandem fusions are the major chromosomal rearrangements underlying the karyotype diversification of muntjacs (Muntiacini) [2–5].

The karyotypes of most Cervidae species have been predominantly studied by standard cytogenetic methods (G-, R-banding, etc.) [3,6,7] and the evolutionary relationships have been studied by comparative chromosome painting in a limited number of cervid species. Cross-species chromosome painting is a variation of the fluorescence in-situ hybridization technique (FISH) and enables us to establish chromosome homology maps, define the sites of chromosome fusions and fissions and to investigate chromosome rearrangements which occurred during karyotype evolution [8,9].

The Capreolinae subfamily consists of nine genera (predominantly with 2n = 70) where chromosomes in only two species were characterized by comparative chromosome painting. Dementeyeva et al. have established the chromosome map of *C*. *pygargus* by cross-species chromosome painting using dromedary probes (*Camelus dromedarius*) [10] and homologies between *Mazama gouazoubira* and Chinese muntjac (*Muntiacus reevesi*) have been established by hybridization with set of Chinese muntjac painting probes [11].

The subfamily Cervinae comprises two tribes Cervini (seven genera) and Muntiacini (two genera). Whereas only Robertsonian translocations were found in karyotypes of the Cervini species, repeated tandem fusions took part in karyotype differentiation of the Muntiacini [2,12]. Generally as the tandem fusions in the karyotype evolution are scarce, the karyotypes of all Muntiacini species were intensively studied by molecular cytogenetic methods based on FISH with painting and BAC probes [4,13,14]. High resolution cross-species comparative mapping between all Muntiacini species were performed, while only two Cervini species were analyzed by chromosomal painting [12].

The subfamily Hydropotinae includes only one species (*Hydropotes inermis*), the most primitive deer, which has retained the karyotype (2n = 70) closely related to the ancestral karyotype of Cervidae. This fact was corroborated by hybridization of painting probes prepared from *M*. *reeve*si [11].

Although the family Bovidae is evolutionally closely related to the Cervidae, cattle painting probes have never been applied to deer chromosomes. The cattle karyotype (2n = 60) is composed of 58 acrocentric autosomes and 2 sex chromosomes and differs only by 1 fission from the Pecoran ancestral karyotype (PAK, 2n = 58)[15,16]. The cattle (*Bos taurus*) fluorescent painting probes have been exploited for comparative karyotype studies not only in many closely related wild animal species [17,18], but also in more phylogenetically distant Cetartiodactyla families [19,20].

Evolutionary rearrangements of ruminant autosomes are rare, except for centric and tandem fusions [18,21–23]. Only the use of high-density BAC (Bacterial Artificial Chromosome) mapping enables precise detection of intrachromosomal rearrangements and their breakpoints in different species. Changes in homologous chromosomes were detected for example in BTA3 homolog between Bovini and Antilopini [24] or in BTA1 homolog between Bovidae and Cervidae, Giraffidae and Antilocapridae [25,19]. An alternative to the high resolution BAC mapping is the *in silico* bioinformatic approach, which allows to detect cryptic chromosomal divergencies. However, this strategy is limited only to the species with sequenced and well assembled genomes [26].

Unlike autosomes, the sex chromosomes differ by more complex chromosomal rearrangements including inversions, centromere shift, heterochromatic variation, and autosomal translocations [22,27–30]. Interspecies variation of X chromosome provides a rich source of phylogenetic information but detailed structure of chromosome X has not been studied in more detail in most Ruminantia species for some considerable time. Just recently, a new investigation of the X chromosome evolution in different representatives of Cetartiodactyla employing high resolution BAC mapping was published [31]. In Cervidae, comparative FISH with whole X chromosome painting probes was employed in several studies [10,12,32]. Better insights into the organization of X chromosomes are enabled by using detailed mapping comparison of BAC or cosmid clones which were used for analysis of the X chromosome rearrangements in various species of the family Bovidae. For instance, Gallagher et al. confirmed the conservation of X chromosome homology regions among members of Bovini, Boselaphini, Tragelaphini, domestic sheep and two deer species using BAC probes [27]. Comparative mapping of three X chromosome-specific bovine cosmids was employed in *Rangifer tarandus* gonosomes [33]. The results of another detailed study concerning conserved syntenies of the X chromosome in four Cervidae species are currently available [31].

A substantial part of the X chromosome can be formed by heterochromatin which can be interspersed or present in blocks in the intercalary or centromeric/pericentromeric regions. The centromeric heterochromatin is mostly composed of satellite DNAs that are also present in centromeres of autosomes. Besides these, the repetitive DNA specific for sex chromosomes can exist, as described in Bovidae [18,24,34] and in Antilocapridae [19]. In Cervidae, an increased size of gonosomes caused by intercalary heterochromatin blocks has been observed in *Rangifer tarandus* and *Elaphodus cephalophus* [12,32].

In the present study, we report a comparative chromosome painting between cattle and various Cervidae species using chromosome painting probes prepared by flow cytometry and laser microdissection. Moreover, we used region specific and BAC probes for more detailed and accurate structural analysis of the autosomal rearrangements and the closer inspection of the structure of the cervid X chromosomes.

## Material and methods

### Ethical statement

All procedures performed in this study were in accordance with the ethical standards of the Veterinary Research Institute (Brno, Czech Republic), which complies with the Czech and European Union Legislation for the protection of animals used for scientific purposes. According to these regulations ethics approval was not required, as the biological material (blood/tissue) was obtained postmortem from animals upon animal slaughter in abattoir or which died during the hunting. The blood from living animals was collected by a ZOO veterinarian during other medical procedures. All collaborating ZOOs have license issued by the Ministry of the Environment of the Czech Republic (Act No 162/2003 Coll.).

### Samples

Whole peripheral blood samples of the 3 specimens of red deer (*Cervus elaphus*, 2n = 68), milu deer (*Elaphurus davidianus*, 2n = 68), rusa deer (*Cervus timorensis russa*, 2n = 60), Eld's deer (*Rucervus eldii*, 2n = 58), moose (*Alces alces*, 2n = 68), reindeer (*Rangifer tarandus*, 2n = 70), roe deer (*Capreolus capreolus*, 2n = 70), fallow deer (*Dama dama*, 2n = 68), Chinese muntjac (*Muntiacus reevesi*, 2n = 46) and giraffe (*Giraffa camelopardalis*; 2n = 30) were obtained from captive born animals held in the Czech zoological gardens and a private deer farm (Bila Lhota, Czech Republic) (Table 1). Blood samples were cultured, harvested and fixed according to standard protocols as described previously [35]. Metaphase chromosome spreads for laser microdissection as well as slides for FISH analysis were prepared according to the procedures described previously [36]. For the assessment of deer karyotypes, GTG-banding was performed using the standard trypsin/Giemsa procedure [37].

**Table 1:** Summary of chromosomal data in the nine Cervidae species enrolled in this study.

Species		2n	FNa	A	M/SM	X	Fusion				Fision
							tandem		centric		
Fallow deer	*Dama dama*	68	68	64	2	A	28/26		17/19		1R; 2; 5; 6; 8; 9
Red deer	*Cervus elaphus*	68	68	64	2	A	28/26		17/19		
Milu deer	*Elaphurus davidianus*	68	68	64	2	A	28/26		17/19		
Eld's deer	*Rucervus eldii*	58	68	44	12	A	28/26		17/19	18/1prox	
									2dist/7	22/1dist	
									5dist/8prox	5prox/10	
Rusa deer	*Cervus timorensis russa*	60	68	48	10	A	28/26		17/19	5prox/22	
									2dist/7	18/3	
									5dist/8prox		
Muntjac	*Muntiacus reevesi*	46	44	44	0	A	5dist/3/7	13/28/26/25/18			
							2prox/9/2dist/11	24/22/5prox			
							29/16	8dist/27			
Roe deer	*Capreolus capreolus*	70	68	68	0	SM	28/26				
Reindeer	*Rangifer tarandus*	70	70	66	2	M	28/26				1; 2; 5; 6; 8; 9
Moose	*Alces alces*	68	70	62	4	SM	28/26		29/17		

2n = chromosome number; NFa = fundamental number of autosomal arms; A = number of acrocentric chromosomes; M/SM = number of sub/metacentric chromosomes; X = X chromosome morphology. The numbers in fusion and fission columns correspond to the bovine chromosome equivalents.

Tissue cultures from cattle (*Bos taurus*, 2n = 60), which were used for flow sorting of chromosomes, were established in our laboratory according to [20].

### Painting probes

Whole chromosomes or chromosomal regions for the construction of painting probes were isolated by flow sorting using MoFlo XDP Cell Sorter (Beckman Coulter, USA) [20] or microdissected by PALM Microlaser system (Carl Zeiss MicroImaging GmbH, Munich, Germany). The chromosomal DNA was then amplified by DOP-PCR (degenerate oligonucleotide primed polymerase chain reaction) [38]. Probe labelling was performed during the secondary PCR with Green-dUTP or Orange-dUTP (Abbott, IL, USA) [39].

### BAC clones

BAC clones were selected from the CHORI-240 cattle library on the basis of NCBI Bos_taurus_UMD_3.1.1 Primary Assembly data and obtained from the Children’s Hospital Oakland Research Institute, BACPAC Resources, USA. Genomic BAC DNA was labelled with biotin-16-dUTP or digoxigenin-11-dUTP (Roche, Mannheim, Germany) using BioPrime Array CGH Genomic Labeling Module (Invitrogen, Carlsbad, CA, USA). Wherever it was possible, two to three BAC clones were selected from the same region and used together as a single probe to generate high quality hybridization signals in the phylogenetically more distant species. Table 2 lists the BAC clones used in the study.

**Table 2:** List of the BAC clones used in the study. BAC clones from the bovine CHORI-BAC library CH240 were selected for the detection of BTA1, BTA3 and BTAX chromosomes. Gray coloring marks two or three BAC clones, which were selected from the same region to generate more intense hybridization signal.

Autosomes				X chromosome		
Chromosome region		Cattle location (Mb)	BAC clone	Chromosome region	Cattle location (Mb)	BAC clone
BTA1	prox	1.89–2.09	169D7	BTAXp	4.60–4.82	132J13
		2.08–2.25	106N15		4.83–4.98	116J8
		2.34–2.50	79I11		23.04–23.22	159O16
	dist	140.80–141.00	118J20		27.70–27.95	27F6
		141.73–141.91	150M16		33.77–34.00	67P21
	tel	154.36–154.55	273F5		38.42–38.55	442N4
		155.61–155.83	106D7		38.89–39.05	305C22
BTA3		4.38–4.62	24H18	BTAXqprox	42.81–43.01	436O23
		9.65–9.91	177N15		47.76–47.97	311B9
		15.40–15.64	82P3		55.08–55.25	198N19
		45.64–45.87	127P23		57.73–57.95	93K24
		53.24–53.51	274A19		62.37–62.53	23A23
		59.18–59.39	122B13		62.54–62.70	382D3
		75.49–75.71	178A24		64.52–64.74	258E20
		83.29–83.50	89E23		68.49–68.68	316D2
		91.98–92.23	141P23		71.51–71.72	467K12
		98.75–98.97	106P15	BTAXqdist	74.95–75.12	40H2
		121.07–121.22	250C14		80.55–80.74	412D24
		121.25–121.43	433N8		84.63–84.81	393C3
					84.77–84.92	146E5
					105.46–10.67	56D3
					126.07–126.23	349K22
					136.01–136.17	34D20
					136.19–136.34	315J10
				BTAX PAR	140.11–140.36	302C6
					144.43–144.62	453C5
					148.27–148.47	326C13

### FISH

The analysis was performed following the protocols for FISH with painting or BAC probes. The posthybridization washing, image capture and processing were carried out as described in [36]. The BAC probes labelled with biotin-16-dUTP and digoxigenin-11-dUTP were detected with Avidin-CY3 (Amersham Pharmacia Biotech, Piscataway, NJ, USA) and antidigoxigenin-fluorescein (Roche). For the analysis, a Zeiss Axio Imager Z2 fluorescence microscope equipped with appropriate fluorescent filters was used. Images of well-spread metaphase chromosomes were captured and analysed using ISIS software (MetaSystems, Altlussheim, Germany).

### Isolation of gonosome specific heterochromatin

*R*. *tarandus* chromosome X and Y were obtained by laser microdissection, amplified by DOP-PCR and subsequently cloned into a pDrive vector (Qiagen, Hilden, Germany). Species-specific clones were selected by dot-blot hybridization following the protocol of [34], fluorescently labeled and checked by FISH. Subsequently, plasmid DNA was isolated and sequenced. The resulting sequences were analyzed using BLASTN (Nucleotide collection (nr/nt) in Cetartiodactyla (taxid:91561)), BLAST2, RepeatMasker software and deposited in the GeneBank.

## Results

### Evolutionary rearrangements of Bovidae and Cervidae autosomes

**Fig 1:**
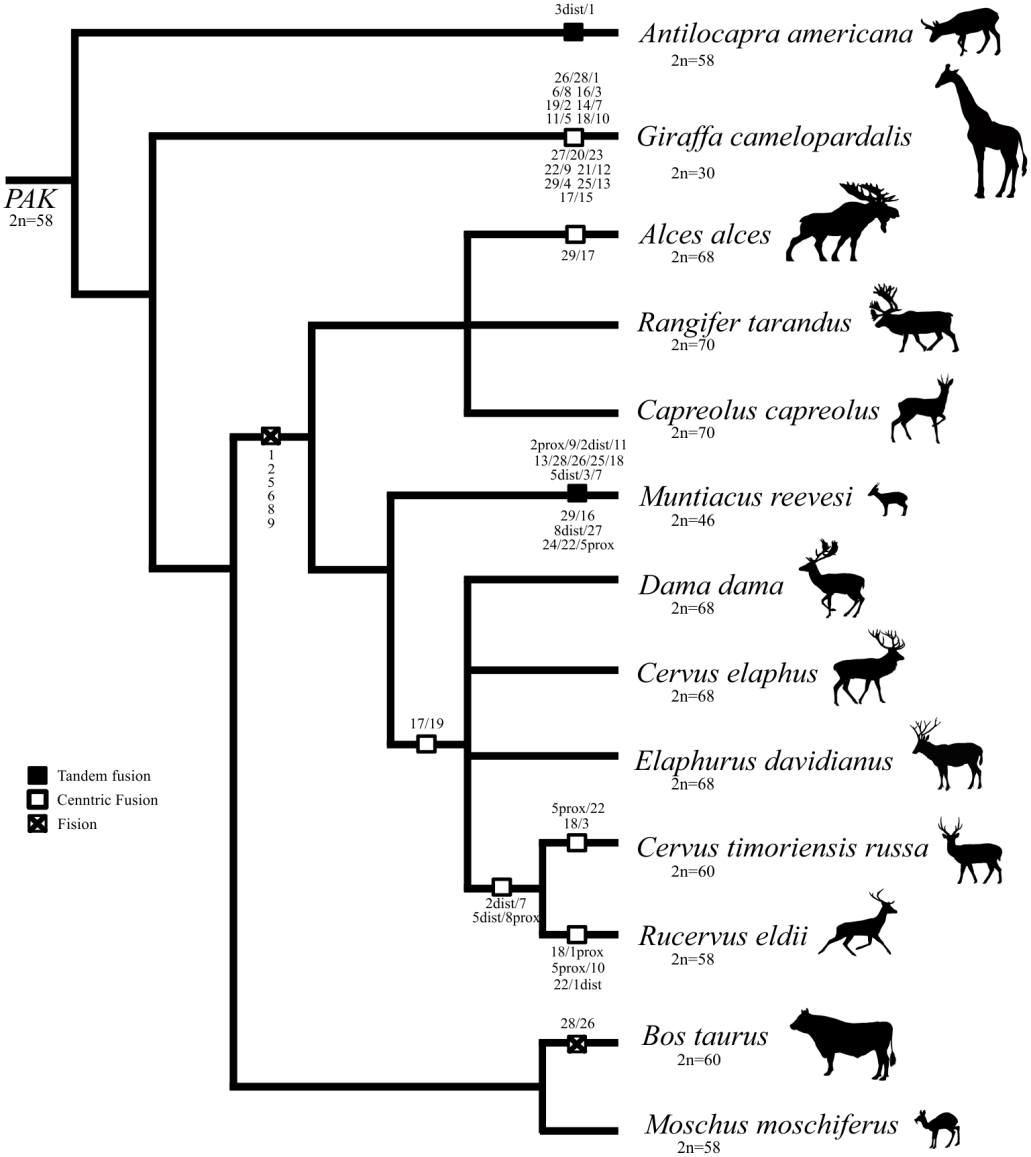
A dendrogram representing phylogenetic relationships between the studied Cervidae species and other Pecoran members. The numbers correspond to the bovine chromosome equivalents. The distances between species are not representative of the evolution time.

To establish the chromosomal homologies between Bovidae and Cervidae families we hybridized painting probes derived from cattle (BTA) on metaphase chromosomes of nine species representing two main subfamilies of Cervidae (Cervinae and Capreolinae) (Fig 1 and Table 1). *E*. *davidianus* (EDA) was chosen as a model representative of family Cervidae whose karyotype has never been studied by molecular cytogenetic methods. Its karyotype is composed of 68 chromosomes, while only one autosomal pair is submetacentric. For the basic homology assessment, we hybridized whole chromosome painting probes (BTA1-BTA29) to chromosomal spreads of EDA. The painting probes from all 29 cattle autosomes revealed 35 conserved chromosomal segments on EDA genome. Only six probes for entire individual cattle chromosomes BTA1, 2, 5, 6, 8 and 9 each hybridized to two separate EDA chromosomes. Cattle chromosomes 26 and 28 are tandemly fused into one acrocentric chromosome, whereas BTA17 and BTA19 correspond to the arm of the sole submetacentric chromosome in *Elaphurus* genome. Each of all other cattle probes had homology only to one EDA chromosome. For detailed description of deer orthologs which represent parts of cattle chromosomes, distal region-specific probes were used. Distal region-specific probes also enabled us to distinguish between tandem and centric fusions in other studied species.

**Fig 2:**
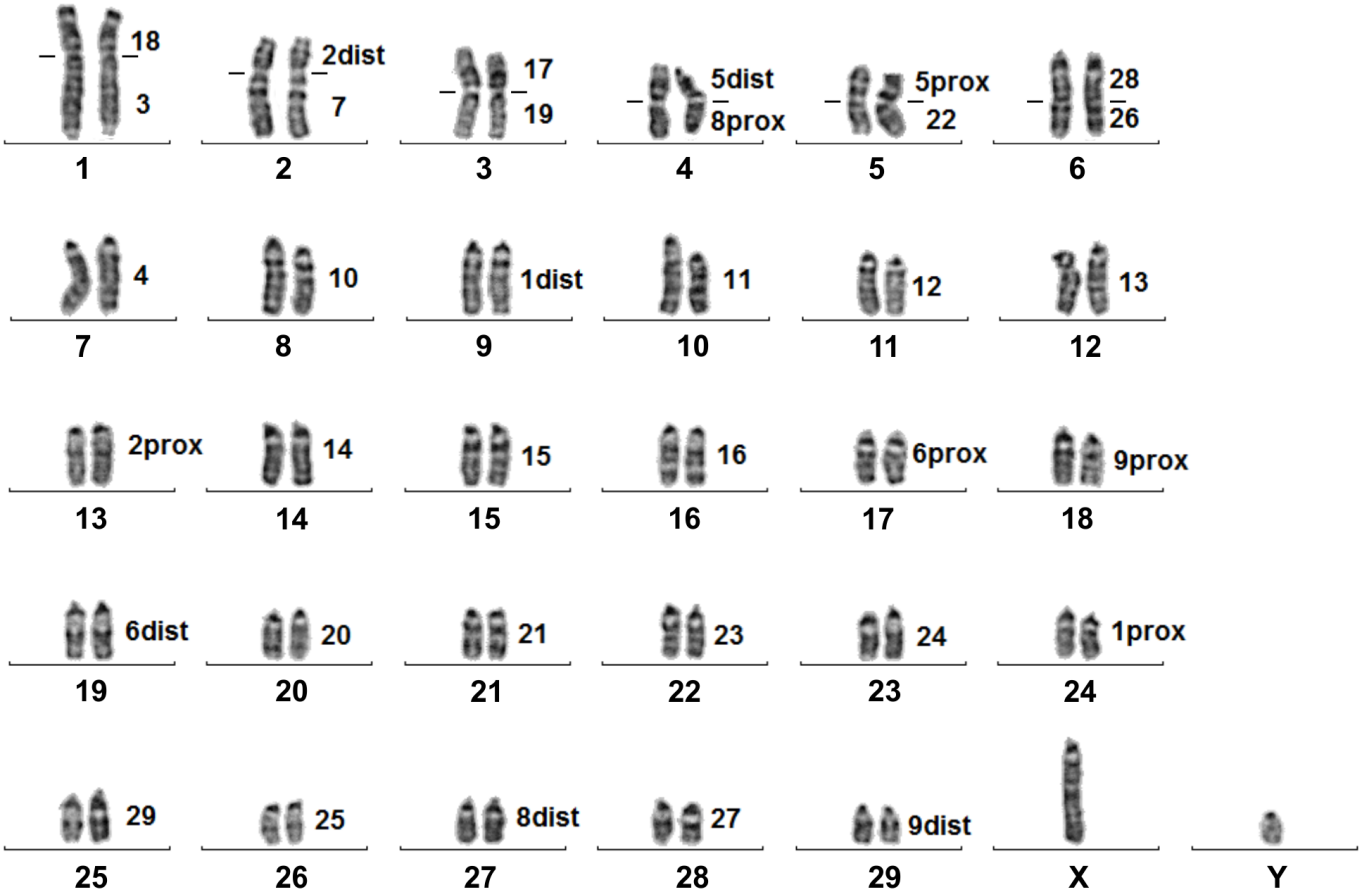
G-banded karyotype of the rusa deer (*Cervus timorensis russa*) with chromosome homologies to the cattle (*Bos taurus*, BTA). Lines on the sides of CTR1-6 chromosome indicate boundaries between two cattle probes.

In another two Cervini species, *C*. *elaphus* (CEL) and *D*. *dama* (DDA), the hybridization of BTA1, 2, 5, 6, 8, 9, 17, 19, 26 and 28 painting probes on appropriate metaphase spreads revealed no obvious differences in their karyotypes in comparison with the EDA. Rusa deer (*C*. *timorensis russa*, CTR) and Eld's deer (*R*. *eldii*, REL) belong as well to the tribe Cervini and they share evolutionary chromosome changes as mentioned in other Cervinae (fusions of BTA17/19 and BTA26/28). However, their chromosome number is lowered due to the other centric fusions. Fusions of the chromosomes BTA2dist/7 and BTA5dist/8prox are shared in both species, whereas fusions BTA18/3, BTA5prox/22, and BTA18/1prox, BTA5prox/10, BTA22/1dist are exclusive to CTR and REL, respectively (Fig 1 and Table 1). G-banded karyotype of CTR with cattle chromosomal homologies is displayed in Fig 2.

**Fig 3:**
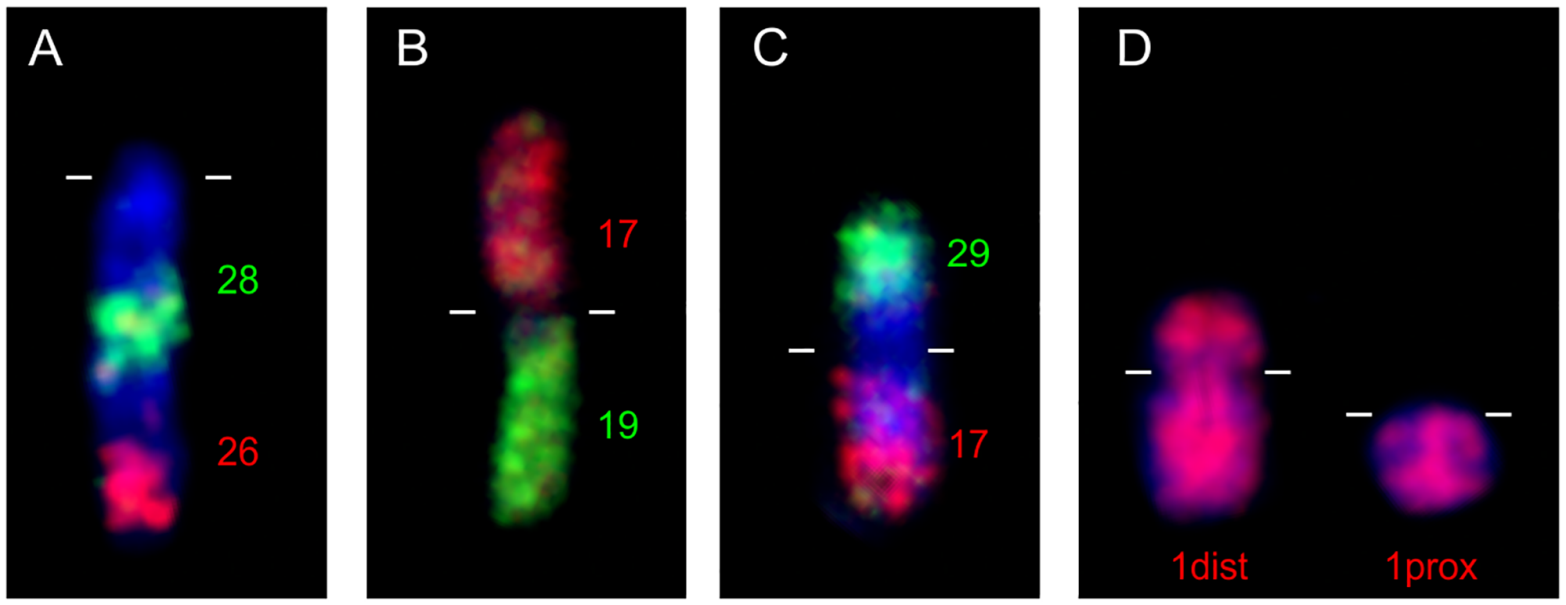
FISH examples demonstrating evolutionary rearrangements between cattle and various Cervidae species. **(A)** Tandem fusion of BTA28/26 in milu deer (EDA) detected by region specific painting probes BTA26dist (red) and BTA28dist (green) **(B)** Centric fusion of BTA17 (red) and 19 (green) in rusa deer (CTR). **(C)** Centric fusion of BTA29/17 in moose (AAL) validated by probes BTA29dist (green) and BTA17dist (red). **(D)** Hybridization of BTA1 on reindeer (RTA) submetacentric and acrocentric orthologs. Centromeres are marked by lines.

Three studied Capreolinae species differ in their karyotypes. The karyotype of *C*. *capreolus* (CCA) is composed of 68 acrocentric autosomes, whereas *R*. *tarandus* (RTA) has, besides acrocentric chromosomes, also one small submetacentric autosome pair and *A*. *alces* (AAL) has similar karyotype as RTA with another larger submetacentric pair. In all the above mentioned Capreolinae species we detected FISH signals of BTA1, 2, 5, 6, 8 and 9 on two separate chromosomes and tandem fusion of BTA28/26. The small submetacentric chromosome in RTA and AAL is homologous to the distal two-thirds part of BTA1, whereas the larger submetactric chromosome in AAL is a result of the centric fusion of BTA29/17. Examples of fluorescence in situ hybridization are displayed in Fig 3.

**Fig 4:**
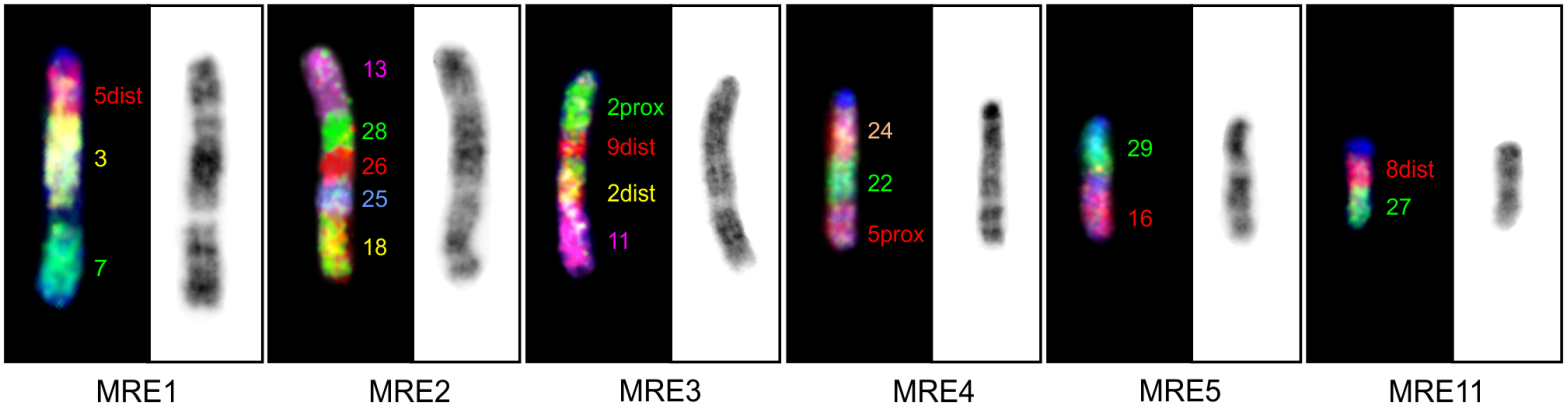
Rearrangements on Chinese muntjac chromosomes MRE1–5 and 11 are demonstrated by hybridization patterns of appropriate cattle painting probes (on the right).

The genus *Muntiacus* is famous for its high degree of interspecific karyotype diversity caused by numerous tandem fusions of ancestral cervid chromosomes. Using cattle painting probes we analyzed selected chromosomes of *M*. *reevesi* in which tandem fusions of cattle orthologs occurred during evolution. Hybridization results and order of cattle homologs on MRE1, 2, 3, 4, 5 and 11 are shown in Fig 4. Each of BTA 1, 5, 6, 8 and 9 probes always hybridized as two homologous blocks on different chromosomes in MRE genome. The BTA2 probe gave a fluorescence signal on MRE3 chromosome however, the signal was split into two blocks separated by BTA9 signal.

**Fig 5:**
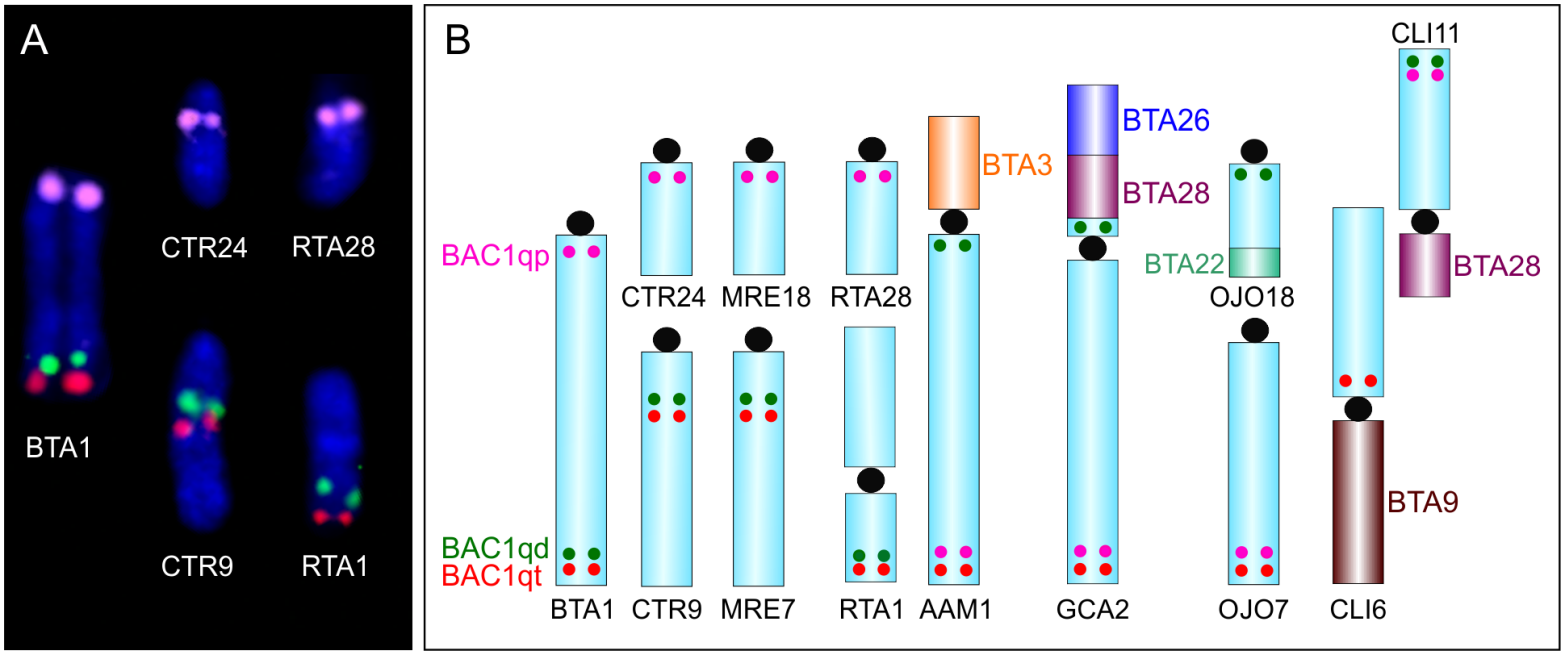
FISH showing hybridization of BTA1 BAC probes and their schematic illustration in various Cetartiodactyl species. **(A**) Hybridization of cattle probes BAC1qp (pink), BAC1qd (green), and BAC1qt (red) probes on chromosomes of cattle (BTA), rusa deer (CTR) and reindeer (RTA). **(B**) Schematic illustration demonstrating rearrangements of BTA1 orthologs in rusa deer (CTR), Chinese muntjac (MRE), reindeer (RTA), pronghorn (AAM), giraffe (GCA), okapi (OJO) and pygmy hippo (CLI). The dots approximate the positions of BAC1qp (pink), BAC1qd (green), and BAC1qt (red) probes.

To find possible intrachromosomal rearrangements on autosomes we selected BAC probes derived from BTA1 and BTA3. Using twelve BAC clones for eleven different loci derived from BTA3 chromosome in EDA, CCA and MRE, we detected the same order of BAC probes as in the cattle. On the other hand, the ortholog of BTA1 in Cervidae is not only split into two separate chromosomes, but the larger of them is intrachromosomaly rearranged. BAC clones derived from the proximal parts of BTA1 (BAC1prox) hybridized to the proximal parts of the smaller ortholog, whereas BAC clones from distal (BAC1dist) and telomeric (BAC1tel) parts of BTA1 hybridized to the middle of the larger acrocentric ortholog (in EDA, DDA, CEL, CTR, REL, CCA and MRE) or to the distal (in relation to the centromere) part of the p-arm of submetacentric chromosome in AAL and RTA. Nonetheless, the position of BAC1dist and BAC1tel between acrocentric and submetacentric chromosomes is altered (Fig 5A).

### Evolutionary rearrangements of the X chromosomes in Bovidae and Cervidae

**Fig 6:**
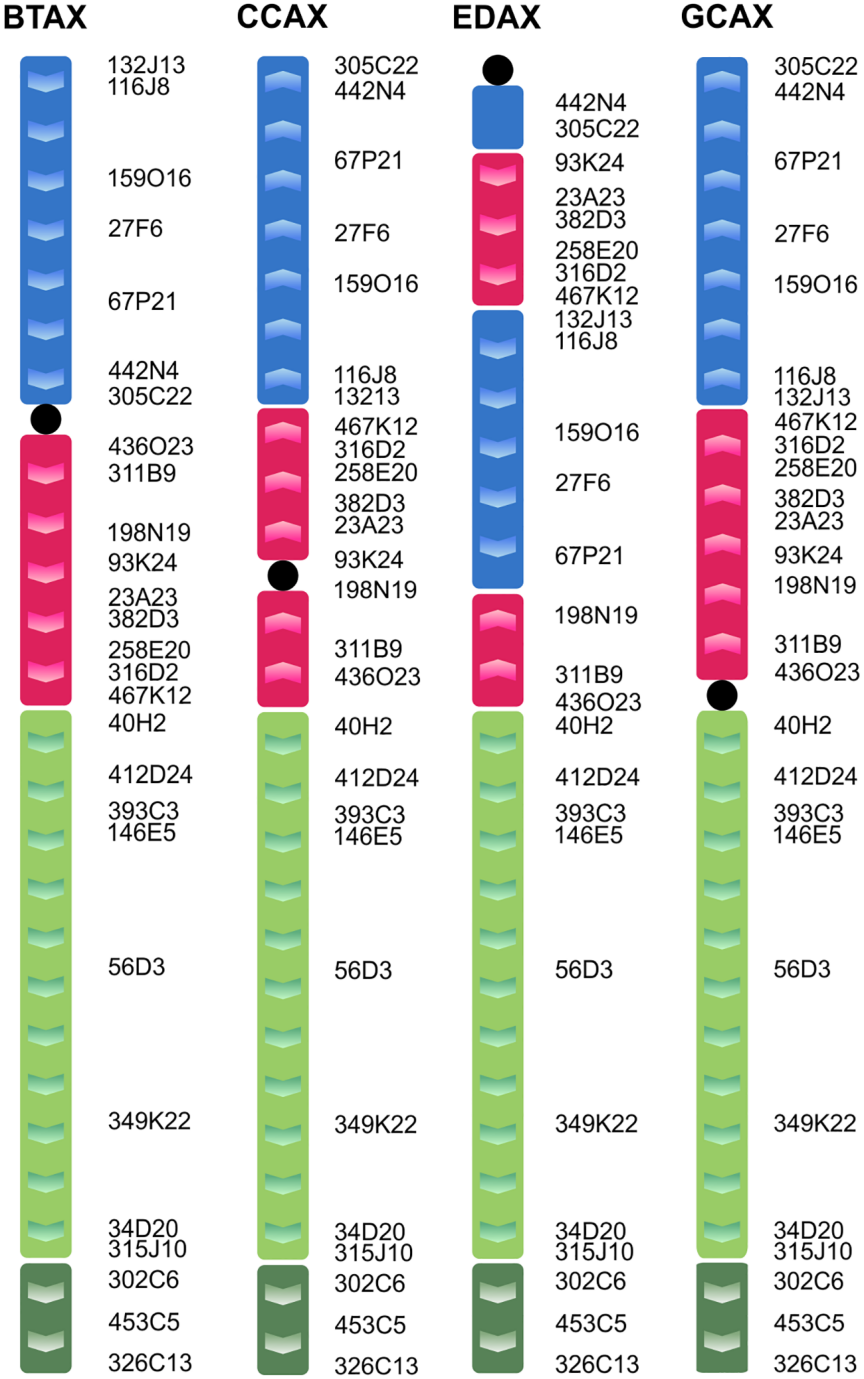
Schematic illustration showing X chromosome segments in cattle (BTA) and their counterparts in roe deer (CCA), milu deer (EDA) and giraffe (GCA). The cattle BAC probes were divided into four groups marked with different colours. Positions of the BAC clones are on the side of the chromosome.

For better depiction of X chromosome rearrangements, we used appropriate BAC clones derived from BTAX. The used BAC X clones were divided into four groups according to their location in cattle submetacentric X chromosome (BTAXp, BTAXqprox, BTAXqdist and BTAXPAR) (Table 2). In the studied Cervidae species, we observed morphologically two different types of X chromosomes, acrocentric type, which is present in Cervinae and a sub/metacentric one in Capreolinae. Using appropriate BAC clones, we also revealed two types of cervid X chromosome. The BTAXqdist and BTAXPAR regions always form one block and they are in the same orientation in acrocentric and submetacentric X variants and they are even conserved within Bovidae and Cervidae. In Capreolinae, the BTAXp and BTAXqprox regions form two blocks and they are separately inverted in comparison to cattle X. The acrocentric variant is much more complex, both BTAXp and BTAXqprox regions are rearranged (Fig 6). The BTAXp block is divided into two separate regions with a breakpoint site between BAC clones 67P21 and 442N4 (34–38 Mb on cattle X). Similarly, BTAXqprox is also separated in two regions with a breakpoint site between BACs 198N19 and 93K24 (55–57 Mb on cattle X).

### Isolation of gonosome specific heterochromatin

**Fig 7:**
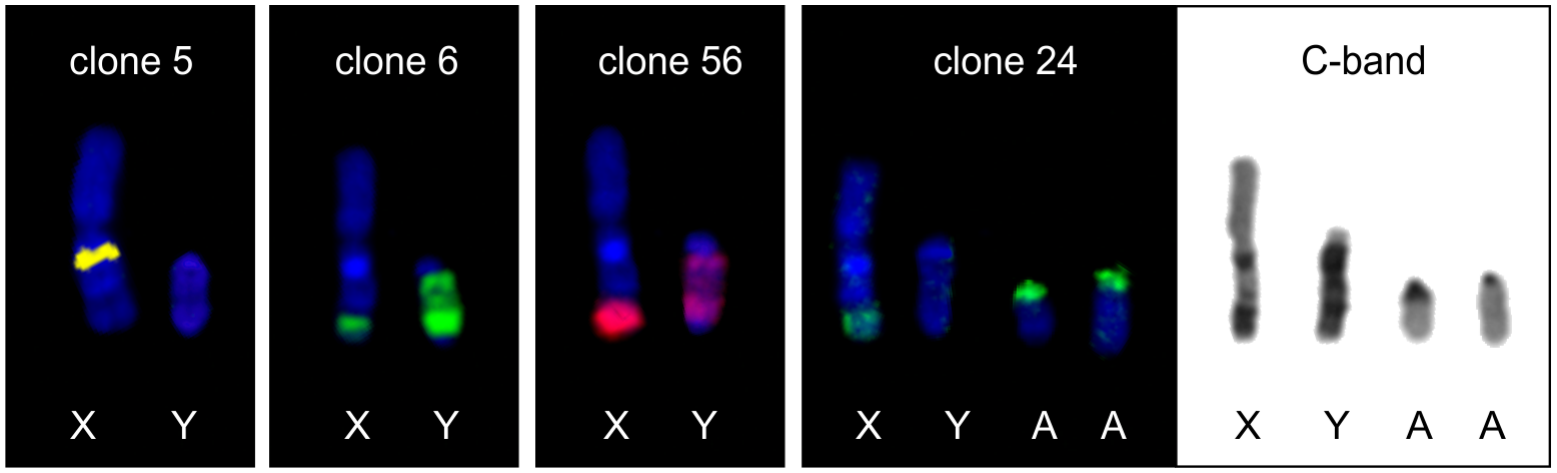
Hybridization results of heterochromatin specific clones on reindeer (RTA) chromosomes. (X, Y—gonosomes; A-autosome).

We prepared four different plasmid clones for heterochromatin regions present on sex chromosomes of RTA. Two of the obtained clones (6 and 56) gave hybridization signal on both X and Y chromosomes. Both clones gave the same overlapping signal on the Y chromosome, whereas at the end of q-arm of X chromosome clone 56 hybridized more proximally than clone 6. Clone 5 was localized in one large block under the centromeric region of the X chromosome. Clone 24 hybridized at the end of the q-arm of X chromosome and in the centromeric regions of most autosomes. Hybridization results are shown in Fig 7. No fluorescence signal was observed when these probes were hybridized on metaphase spreads of related species (ALL).

The sequences of all clones were deposited in NCBI. No significant homologies between clone sequences were found using BLAST2 program. RepeatMasker revealed internal repeat only in clone 6. Three of the clones showed a similarity to different parts of the 135Mb long *Ovis canadensis* chromosome X sequence (CP011912) (Table 3).

**Table 3:** Isolated and characterized clones obtained from reindeer (RTA) gonosomes.

Clone no.	NCBI ID	Hybridization	Repeat Masker	BLAST
5	MF461211	X	No repetitive sequences	CP011912–82%
6	MF461212	X, Y	Simple repeat (ATCAC) 18%	CP011912–81%
24	MF461214	X, centromeres	No repetitive sequences	No similarity
56	MF461213	X, Y	No repetitive sequences	CP011912–77%

## Discussion

In this work, we provide the first genome-wide investigation of the karyotypes of various cervid species by cross-species FISH using cattle painting probes. Although the family Bovidae is in evolutionary terms a closely related group to the Cervidae, comparative evolutionary studies based on reciprocal chromosome painting are limited. Only Burkin et al. hybridized all sheep (*Ovis aries*) chromosome painting probes on chromosomes of *Muntiacus muntjak vaginalis* and Chi *et* al. applied whole chromosome probes from *M*. *reevesi* on metaphases of gayal (*Bos frontalis*) [40,41].

Our comparative chromosomal analysis confirmed that common karyotype differences between cattle and studied Cervidae species includes disruptions of orthologs of BTA1, 2, 5, 6, 8, 9 and tandem fusion BTA28/26. Whereas fissions of six cattle orthologs are characteristic evolutionary changes in Cervidae, the fusion BTA28/26 is typical for all pecoran species, excluding Bovidae. The same conclusions were deduced by zoo-FISH using dromedary probes (*Camelus dromedarius*) on Siberian roe deer (*Capreolus pygargus*) [10].

The main characteristic in all studied Cervini species is one common submetacentric chromosome which arose via Robertsonian fusion of segments homologous to the BTA17 and 19. This is in accordance with previous studies, based on G, R—banded chromosomes [3,7,25]. We confirmed centric fusions of cattle homologs in CTR and REL biarmed chromosomes, which were previously described by R-banding and BAC mapping [3].

From Capreolinae, we studied three species, where two of them (AAL and RTA) had a small metacentric chromosome in their karyotype. This submetacentric is not formed by centric fusion of small acrocentric ancestral chromosomes, but it originated from distal two-thirds of BTA1 homolog as in other cervid species, where it occurs in acrocentric form. In addition to this small submetacentric, another larger submetacentric chromosome is present in AAL genome. This chromosome arose from centric fusion of BTA29/17.

The tribe Muntiacini is well known for its high degree of interspecific karyotype diversity between closely related species, which is caused by numerous tandem fusions. For instance, *M*. *muntjak vaginalis* has the lowest chromosome number known in mammals (2n = 6/7), whereas *M*. *reevesi* (another morphologically similar species used in this study) has the highest diploid number (2n = 46) of all *Muntiacus* species [42]. More than one cattle painting probe hybridized on six pairs of MRE chromosomes. Number of synteny blocks on these six pairs of chromosomes ranged from two (MRE5, MRE11) to five (MRE2) (Fig 4). We confirmed that each of the orthologs of BTA1, 2, 5, 6, 8 and 9 always create two separate homologous blocks in MRE genome, although both BTA2 blocks are present on the MRE3 chromosome. Our obtained hybridization data are mostly in accordance with the results of Chi et al. who used probes from MRE on gayal (*B*. *frontalis*) metaphase chromosomes [41]. The only difference was found on MRE3 chromosome, where we distinguished two separated homologous blocks of BTA2, which cannot be revealed with the use of MRE probes. In MRE, the ancestral tandem fusion BTA28/26 is not present as a separate chromosome, but it forms one block on MRE2.

Evolutionary intrachromosomal rearrangements in ruminant autosomes are rare. However, the changes on homologs of BTA3 and BTA1 were detected in Antilopini, Giraffidae, Cervidae and Antilocapridae [19,24,25]. Therefore, we used BAC probes derived from BTA1 and BTA3 to detect possible intrachromosomal rearrangements in selected Cervidae species. Despite using high density BAC mapping on the BTA3 ortholog in studied Cervidae species, no changes in the order of BAC clones were revealed.

On the other hand, on the ortholog of BTA1 in all studied Cervidae species the complex rearrangements were detected. Those findings are not so surprising, because BTA1 orthologs are very diverse in many species due to the disruptions, inversions, and translocations. In all our nine Cervidae species, the BTA1 ortholog is split into two chromosomes of different sizes. The larger chromosome is acrocentric in most species with the exception of AAL and RTA where due to the centromere shift it has submetacentric form. The state of BTA1 orthologues in AAL and RTA represents simple disruption into two chromosomes with retained BAC order. The situation with BTA1 ortholog in EDA, DDA, CEL, CTR, REL, CCA and MRE is more complex. In these species, rearrangement of the larger ortholog representing two-thirds of BTA1 occurred (Fig 5A). This rearrangement of the BTA1 ortholog was previously described in Vietnamese sika deer (*Cervus nippon*) using a different set of BAC probes [25]. Another structure of the BTA1 ortholog is present in the genome of giraffe (*Giraffa camelopardalis*), pronghorn (*Antilocapra americana*) and okapi (*Okapia johnstoni*), where the BTA1 equivalent has been disrupted and inverted [19]. Outside the suborder Ruminantia, the BTA1 ortholog rearrangements were detected in pygmy hippo (*Choeropsis liberiensis*, CLI) from the family Hippopotamidae. The ortholog of BTA1 in pygmy hippo is split into two chromosomal arms (CLI6p, CLI11q) with other multiple rearrangements [20]. All mentioned BTA1 ortholog changes are summarized and illustrated in Fig 5B. Previous studies, which used dromedary and human painting probes, found that Ruminantia and Pecora ancestral characteristics of the BTA1 ortholog are retained in the giraffe and pronghorn chromosomes GCA2 and AAM1 [16,43]. From that ancestral state, one fission and one inversion occurred in the evolutionary line leading to Bovidae, Moschidae and Cervidae. In Cervidae, another fission of BTA1 ortholog formed two separate chromosomes (RTA and AAL) and subsequently in Cervinae and CCA another break and translocation occurred on the larger of them. Considering complexity of rearrangements of BTA1 ortholog in different species (Fig 5B), the employment of the high density BAC mapping could reveal progressive chromosomal changes which occurred during evolution. The increased rates of chromosomal rearrangements in some Pecoran chromosomes suggest the presence of the breakpoint hotspots that can explain such increased levels of rearrangements on these particular chromosomes [44,45].

Unlike autosomes, the sex chromosomes in Cetartiodactyla are variable between species due to the complex chromosomal changes including inversions, centromere shift and heterochromatic variation [22,30]. The X chromosome rearrangements have not been studied in more detail in most Cervidae species even though they proved to be a rich source of phylogenetic information [46]. For better depiction of X chromosome rearrangements, we used appropriate BAC clones (Table 2). Two different morphological types of X chromosomes were observed in studied Cervidae species, acrocentric type present in Cervinae and submetacentric one in Capreolinae. Both types of X differed not only by morphology, but also by the order of used BAC clones. The structure of the submetacentric variant is closer to the BTAX, whereas the acrocentric type is much more rearranged (Fig 6).

In Cervidae, the number of papers using Bovidae X chromosome BAC/cosmid clones is limited. Only a few cosmid clones were hybridized on the X chromosome of RTA and several BAC clones on two Cervidae species (*C*. *nippon* and *Odocoileus virginianus*), which are carriers of both X chromosome variants [27,33]. Recently, the structure of the X chromosome was described in four Cervidae species (*Capreolus pygargus*, AAL, DDA and EDA) by high resolution BAC mapping [31]. Our hybridization data are mostly in agreement with the above mentioned papers and substantially extend knowledge concerning the structure of cervid X chromosomes. In addition, our selection of X BAC clones enabled us to detected not only four, but five synteny blocks on the X chromosome of DDA that were not previously revealed by other BAC mapping [31]. We observed the same structure of X chromosome with five synteny blocks in all six studied Cervinae species (DDA, CEL, EDA, CTR, REL and MRE). On the other hand, Proskuryakova et al. studied only two Cervinae species (DDA and EDA) and they described two different types of X chromosome rearrangements in those two closely related animals. Studies that describe the detailed structure and rearrangements of the X chromosome outside the family Bovidae were scarce and limited only to a few species from the Giraffidae, Cervidae, Antilocapridae and Hippopotamidae [19,20,27]. Recently, new investigation of the X chromosome structure in eighteen different Cetartiodactyla species using comparative BAC mapping appeared [31]. Taking into account all known structures of X chromosomes in different species, the acrocentric (Cervinae) variant is unique, whereas the submetacentric (Capreolinae) type is similar to the X chromosome of the giraffe and hippo (without regard to the centromere position). For confirmation of the similarity between Capreolinae and giraffe X chromosome structure we hybridized the same set of BAC probes on giraffe chromosomes (Fig 6). The order of BAC clones on giraffe X was same as in *Capreolus*. With regard to those facts, the X chromosome structure present in Capreolinae, Giraffidae and Hippopotamidae appears to be the ancestral type. Those findings are in agreement with the most recently published data in which authors postulate that the Pecoran ancestral X chromosome (PAX) is preserved in the genomes of giraffe and moose and differs from the Cetartiodactyla ancestral X chromosome (CAX) only by centromere position [31].

Rearrangements on X chromosomes can be caused by the presence of heterochromatin blocks, which are composed of repetitive sequences. The occurrence of those sequences was described in Bovidae [18,24,34] and Antilocapridae [19]. Among Cervidae species, such heterochromatin blocks have only been observed in *R*. *tarandus* and *Elaphodus cephalophus*, which are responsible for the increased size of gonosomes [12,32]. In RTA, several heterochromatin motives were characterized by *in situ* hybridization of microdissected X chromosome regions [32]. In our study, we also used the microdissection method for generation of heterochromatin specific clones with the subsequent FISH localization and Sanger sequencing. Similarly to [32], we characterized several variants of heterochromatin repeats in RTA with different hybridization patterns. Although all examined clones were short (101–447 bp), they gave strong fluorescent signals confirming their repetitive character. Internal tandem repeat motif was not found in any of the studied clones, thus, the obtained sequence cannot be characterized as microsatellites. We confirmed that these repetitive sequences are characteristic for RTA as they do not hybridize to metaphase chromosomes of AAL.

## Conclusion

In this study, we provide comparative chromosome painting between various Cervidae species and cattle. For the first time, we report in-depth examination of *C*. *timorensis russa*, *E*. *davidianus*, *R*. *eldii*, *A*. *alces* and *R*. *tarandus* karyotypes. We also carried out a detailed and accurate structural analysis of the cervid X chromosomes using bovine BAC clones. Heterochromatic blocks causing elongation of *R*. *tarandus* gonosomes were studied to the sequence level.

## Abbreviations

AAL: moose (*Alces alces*)
AAM: pronghorn (*Antilocapra americana*)
BAC: Bacterial Artificial Chromosome
BTA: cattle (*Bos taurus*)
CAX: Cetartiodactyla ancestral X
CCA: roe deer (*Capreolus capreolus*)
CEL: red deer (*Cervus elaphus*)
CLI: pygmy hippo (*Choeropsis/Hexaprotodon liberiensis*)
CTR: rusa deer (*Cervus timorensis russa*)
DDA: fallow deer (*Dama dama*)
dist: distal in relation to the centromere
EDA: milu deer (*Elaphurus davidianus*)
FISH: fluorescence in situ hybridization
GCA: giraffe (*Giraffa camelopardalis*)
MRE: Chinese muntjac (*Muntiacus reevesi*)
OJO: okapi (*Okapia johnstoni*)
PAK: Pecoran ancestral karyotype
PAR: pseudoautosomal region
PAX: Pecoran ancestral X
prox: proximal in relation to the centromere
RAK: Ruminantia ancestral karyotype
REL: Eld's deer (*Rucervus eldii*)
RTA: reindeer (*Rangifer tarandus*)
